# O-GlcNAcylation mediates metastasis of cholangiocarcinoma through FOXO3 and MAN1A1

**DOI:** 10.1038/s41388-018-0366-1

**Published:** 2018-06-18

**Authors:** Chatchai Phoomak, Atit Silsirivanit, Dayoung Park, Kanlayanee Sawanyawisuth, Kulthida Vaeteewoottacharn, Chaisiri Wongkham, Eric W.-F. Lam, Chawalit Pairojkul, Carlito B. Lebrilla, Sopit Wongkham

**Affiliations:** 1 Department of Biochemistry, Faculty of Medicine, Khon Kaen University, Khon Kaen, 40002 Thailand; 2 Cholangiocarcinoma Research Institute, Khon Kaen University, Khon Kaen, 40002 Thailand; 3 Department of Chemistry, University of California, Davis, CA 95616 USA; 4 Department of Surgery, Beth Israel Deaconess Medical Center, Harvard Medical School, Boston, MA 02115 USA; 5 Department of Surgery and Cancer, Imperial Centre for Translational and Experimental Medicine, Imperial College London, London, W12 0NN UK; 6 Department of Pathology, Faculty of Medicine, Khon Kaen University, Khon Kaen, 40002 Thailand

## Abstract

The leading cause of death in cancer patients is metastasis, for which an effective treatment is still necessary. During metastasis, cancer cells aberrantly express several glycans that are correlated with poor patient outcome. This study was aimed toward exploring the effects of O-GlcNAcylation on membranous N-glycans that are associated with the progression of cholangiocarcinoma (CCA). Global O-GlcNAcylation in CCA cells was depleted using specific siRNA against O-GlcNAc transferase (OGT), which transfers GlcNAc to the acceptor proteins. Using an HPLC-Chip/Time-of-Flight (Chip/TOF) MS system, the N-glycans associated with O-GlcNAcylation were identified by comparing the membranous N-glycans of siOGT-treated cells with those of scramble siRNA-treated cells. In parallel, the membranous N-glycans of the parental cells (KKU-213 and KKU-214) were compared with those of the highly metastatic cells (KKU-213L5 and KKU-214L5). Together, these data revealed that high mannose (Hex_9_HexNAc_2_) and biantennary complex (Hex_5_HexNAc_4_Fuc_1_NeuAc_1_) N-linked glycans correlated positively with metastasis. We subsequently demonstrate that suppression of O-GlcNAcylation decreased the expression of these two N-glycans, suggesting that O-GlcNAcylation mediates their levels in CCA. In addition, the ability of highly metastatic cells to migrate and invade was reduced by the presence of *Pisum Sativum* Agglutinin (PSA), a mannose-specific lectin, further indicating the association of high mannose type N-glycans with CCA metastasis. The molecular mechanism of O-GlcNAc-mediated progression of CCA was shown to proceed via a series of signaling events, involving the activation of Akt/Erk (i), an increase in FOXO3 phosphorylation (ii), which results in the reduction of MAN1A1 expression (iii) and thus the accumulation of Hex_9_HexNAc_2_ N-glycans (iv). This study demonstrates for the first time the association between O-GlcNAcylation, high mannose type N-glycans, and the progression of CCA metastasis, suggesting a novel therapeutic target for treatment of metastatic CCA.

## Introduction

Metastasis, a progressive process in which cancer cells develop the ability to colonize distant organs, is responsible for the majority of cancer deaths [1]. The occurrence of metastasis is particularly high for cholangiocarcinoma (CCA), a cancer of the bile duct. CCA is highly prevalent in southeast Asia and ranks among diseases with the highest mortality rates, following HIV and stroke [2–4]. At present, few effective treatments for advanced CCA outside of surgical resection is available to prolong the survival of the patients.

Modification of proteins via glycosylation is one of the most common post-translational processes that exercises key roles in homeostatic functions, e.g., inflammation, cell–cell interactions, morphogenesis, and immunity. Aberrant glycosylation is commonly found in cancer, some of which associate with metastatic processes [5–7]. The correlation between the membranous glycosylation and the progression of cancer has been collectively reported [8–11]. In CCA, several aberrant glycans and glycoproteins are correlated with CCA progression, such as carbohydrate antigen 19-9, carcinoembryonic antigen, mucin (MUC)1, MUC2, MUC5AC, serum α1β-glycoprotein, and several lectin-binding glycans [12–17].

O-GlcNAcylation is a form of protein modification, where a single moiety of *N*-acetylglucosamine (GlcNAc) is added to the target protein without further elongation or modification into more complex structures [18–20]. Modification with O-GlcNAc is a reversible process, in which GlcNAc is added to or removed from the amino acid Ser/Thr of a protein by O-GlcNAc transferase (OGT), or O-GlcNAcase (OGA), respectively [19]. Alterations of O-GlcNAcylation can dysregulate protein function, disrupting processes such as protein phosphorylation, stability, protein–protein interaction, and protein localization [21]. Accordingly, aberrant O-GlcNAcylation relates to a number of diseases, including cancer [22]. In particular, the increase of O-GlcNAcylation in tumor tissues has been shown to occur with the progression of cancers, including cancers of the breast, colon, liver, lung, pancreas and prostate [23] as well as CCA [24]. In contrast, suppression of OGT in CCA cells could decrease invasion and migratory capabilities [25]. Moreover, we have previously shown that CCA cells with high metastatic abilities have higher O-GlcNAcylation levels than those with low metastasis [26]. Nevertheless, how O-GlcNAcylation modulates progression of CCA metastasis remains unclear.

To our knowledge this study reports for the first time the molecular link between O-GlcNAcylation and membranous high mannose type N-glycans related to cancer progression. Comparing the glycomic data from siOGT treated and control cells with those from high and low metastatic cells, the metastatic-specific membranous N-glycans that were regulated by O-GlcNAcylation were identified. Subsequently, the molecular mechanism of how O-GlcNAcylation regulates the metastasis-related N-glycans were elucidated. These membranous N-glycans could be new prognostic markers or novel therapeutic targets for metastatic CCA.

## Results

### Expression of specific high mannose type N-glycans may be modulated by O-GlcNAcylation

**Fig. 1:**
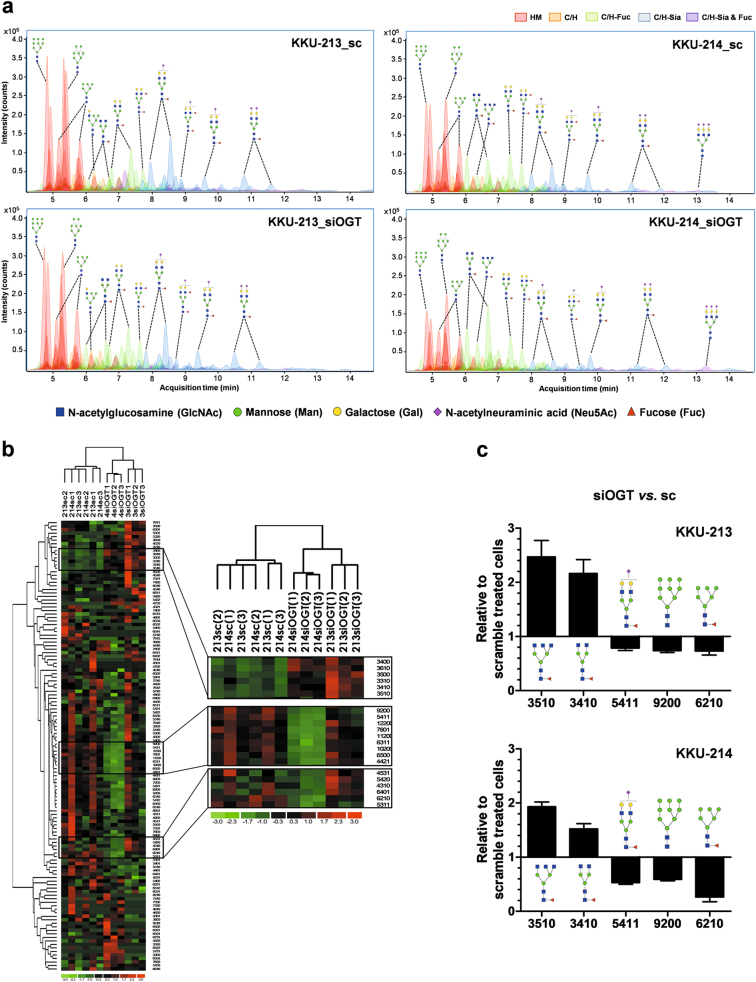
Suppression of O-GlcNAcylation altered N-glycans expression in CCA cells. **a** Quantitative analysis of membranous N-glycans were determined using the Agilent MassHunter Qualitative analysis software. The colors of each peak represent the type of N-glycans; red for high mannose (HM) glycans; orange for undecorated complex/hybrid (C/H) glycans; green for fucosylated complex/hybrid (C/H−F) glycans; blue for sialylated complex/hybrid (C/H−S) glycans; and purple for fucosylated-sialylated complex/hybrid (C/H-FS) glycans. **b** Heat map represented numbers of differentially expressed N-glycans between scramble and siOGT-treated CCA cells (KKU-213 and KKU-214). Triplicate samples were analyzed. **c** The five N-glycans that were differentially expressed in both cell lines when O-GlcNAcylation was suppressed using siOGT

We first investigated the involvement of O-GlcNAcylation in the membranous N-glycan expression. N-Glycan analysis was performed by graphitized carbon column chip-mounted nano-LC-MS. The N-glycan profiles of siOGT and control scramble siRNA (sc) treated cells were determined and compared. LC-MS analysis yielded nearly 130 N-glycans over a dynamic range of four orders of magnitude. The majority of membranous N-glycan on CCA cells were high mannose type N-glycans (Fig. 1a, b and Table S1). A ratio of N-glycan intensity of siOGT to sc higher than 1.2 was classified as up-regulated and a ratio lower than 0.8 were set as down-regulated. There were 16 N-glycans differentially expressed in KKU-213 and 31 in KKU-214 (Table S1). Among these, only five N-glycans were commonly altered when O-GlcNAcylation was suppressed in both KKU-213 and KKU-214 cells (Fig. 1c). Specifically, Hex_9_HexNAc_2_, Hex_6_HexNAc_2_Fuc_1_, and Hex_5_HexNAc_4_Fuc_1_NeuAc_1_ were down-regulated, whereas Hex_3_HexNAc_5_Fuc_1_ and Hex_3_HexNAc_4_Fuc_1_ were up-regulated. The synthesis of these five N-glycans may be associated with O-GlcNAcylation in CCA cells.

### Expressions of specific high mannose type N-glycans associate with metastatic ability of CCA cells

**Fig. 2:**
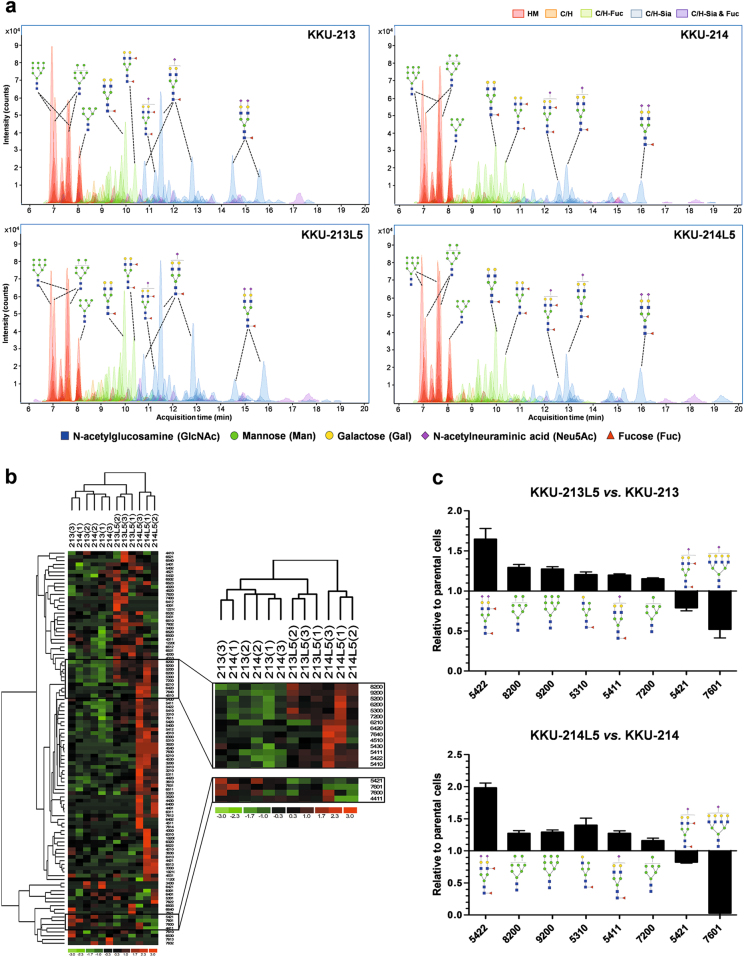
N-glycan profiles of parental and highly metastatic CCA cells. The membranous N-glycans of parental (KKU-213 and KKU-214) and highly metastatic cells (KKU-213L5 and KKU-214L5) were compared. **a** Chromatograms of N-glycans released from CCA cells. The colors of each peak represent the type of N-glycans: red, high mannose (HM) glycans; orange, undecorated complex/hybrid (C/H) glycans; green, fucosylated complex/hybrid (C/H−F) glycans; blue, sialylated complex/hybrid (C/H−S) glycans; purple, fucosylated-sialylated complex/hybrid (C/H-FS) glycans. **b** Heat map represents the numbers of differentially expressed N-glycans between parental and highly metastatic CCA cells. Triplicate samples were analyzed. **c** Given the expression of parental cells (KKU-213 and KKU-214) as 1, eight N-glycans were differentially expressed in both highly metastatic CCA cells

We next sought to identify the membranous N-glycans that may be correlated with metastasis. The expression profiles of the membranous N-glycans from two pairs of CCA cell lines with different metastatic potentials, the parental (KKU-213 and KKU-214) and the highly metastatic (KKU-213L5 and KKU-214L5) cells, were determined. A total of 136 N-glycans were found in both parental and highly metastatic CCA cells with different expression levels (Fig. 2a, b and Supplementary Table S2). There were 20 N-glycans differentially expressed between KKU-213 and KKU-213L5, and 30 N-glycans between KKU-214 and KKU-214L5 (Supplementary Table S2). Of these, eight differentially expressed N-glycans in both KKU-213L5 and KKU-214L5 were revealed. Six N-glycans were up-regulated (Hex_5_HexNAc_4_Fuc_2_NeuAc_2_, Hex_8_HexNAc_2_, Hex_9_HexNAc_2_, Hex_5_HexNAc_3_Fuc_1_, Hex_5_HexNAc_4_Fuc_1_NeuAc_1_, and Hex_7_HexNAc_2_) while two were down-regulated (Hex_5_HexNAc_4_Fuc_2_NeuAc_1_ and Hex_7_HexNAc_6_NeuAc_1_) (Fig. 2c). These eight N-glycans may associate with the metastatic abilities of CCA cells.

### Two membranous N-glycans are associated with metastatic activities and O-GlcNAcylation

**Fig. 3:**
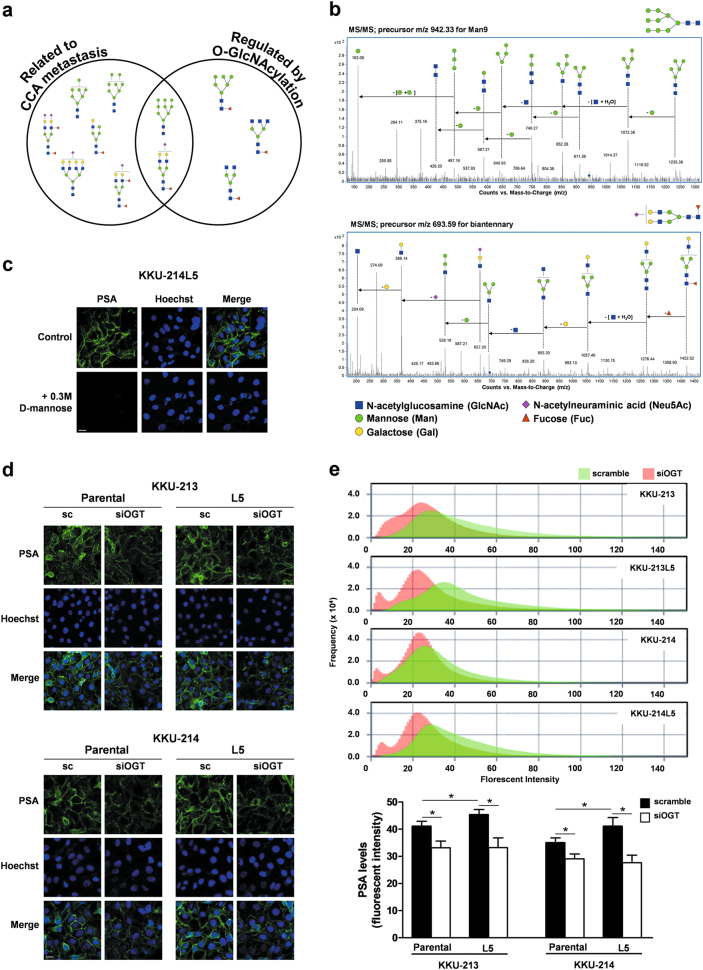
The expression levels of membranous N-glycans were modulated by O-GlcNAcylation. **a** Expression of high mannose type N-glycan, Hex_9_HexNAc_2_ and biantennary complex type N-glycan, Hex_5_HexNAc_4_Fuc_1_NeuAc_1_, were found to be associated with metastatic ability and O-GlcNAcylation. **b** The MS/MS spectra confirmed glycan structures of both N-glycans; high mannose type (*m/z* 942.33) and for biantennary complex type (*m/z* 693.59). **c** PSA-cytofluorescence stained high mannose glycans at the cell membrane. The specificity of PSA to mannosylated glycan was proved by sugar inhibition test. **d** PSA-cytofluorescent staining was reduced in siOGT-treated cells. **e** Histograms and graphs represent the quantitative levels of PSA-fluorescent signals. The results are mean ± SEM of one representative from two independent experiments; *P *<* 0.05, student’s *t* test

To determine the N-glycans that may be involved in metastasis under the modulation of O-GlcNAcylation, the membranous N-glycans associated with O-GlcNAcylation (Fig. 1c) and those associated with metastatic potential (Fig. 2c) were aligned. As shown in Fig. 3a, Hex_9_HexNAc_2_ and Hex_5_HexNAc_4_Fuc_1_NeuAc_1_, two N-glycans were commonly found in both data sets. To confirm the structures of the predicted N-glycans, the structures of Hex_9_HexNAc_2_ and Hex_5_HexNAc_4_Fuc_1_NeuAc_1_ were analyzed using graphitized carbon column chip-mounted nano-LC-MS/MS. The predicted N-glycans were confirmed by MS/MS, as precursor *m/z* 942.33 for Hex_9_HexNAc_2_, a high mannose type N-glycan (Fig. 3b, upper panel) and precursor *m/z* 693.59 for Hex_5_HexNAc_4_Fuc_1_NeuAc_1_, a biantennary complex type N-glycan (Fig. 3b, lower panel).

### Suppression of O-GlcNAcylation reduces expression of membranous high mannose type N-glycans

The high mannose type N-glycan, Hex_9_HexNAc_2_, was the highest abundant in CCA cells (Figs 1a and 2a) and therefore was selected for further study. To confirm the connection between the expression of the high mannose type N-glycans at the cell surface and intracellular O-GlcNAcylation, the expression of high mannose type N-glycans in the siOGT-treated *vs*. scramble siRNA-treated cells were determined. Both *Pisum Sativum* Agglutinin (PSA) and Concanavalin A (Con A) have been used to detect high mannose type N-glycan in several studies [27–30]. Our preliminary study using lectin-histochemistry of PSA and Con A in tumor tissues from CCA patients revealed that PSA but not Con A could differentiate non-metastatic from. metastatic CCA tissues (Supplementary Figure S1). The PSA signal was strongly positive in CCA tissues with metastasis, weaker in non-metastatic tissue, and negative with hepatocytes. Con A, in contrast, reacted strongly with both metastatic and non-metastatic CCA, as well as hepatocytes. On the basis of these observations, we selected to use PSA to detect high mannose type N-glycans in the subsequent study.

High mannose type N-glycans expressed at the cell surface were quantified using cytofluorescent staining of PSA, a mannose binding lectin. The specificity of PSA to mannosylate N-glycan was first determined using a sugar inhibition test. As shown in Fig. 3c, the PSA-cytofluorescent staining was localized at cell membranes. Adding 0.3 M D-mannose to neutralize the binding ability of PSA significantly diminish the staining signal of PSA. PSA-cytofluorescent staining was next performed and compared between the siOGT-treated cells and the scramble siRNA-treated cells. The PSA-cytofluorescent staining signals were reduced in siOGT-treated cells compared with those of scramble control cells (Fig. 3d). Similar patterns were observed for both parental and highly metastatic CCA cells. Quantitative analysis of the cytofluorescent signals are presented in Fig. 3e. The PSA-binding signals were significantly decreased in siOGT-treated cells (*P* < 0.05) and the reduction was more pronounced in the highly metastatic cells compared to the parental cells. These data connect the intracelluar O-GlcNAcylation to the expression of cell surface high mannose type N-glycans. In addition, higher basal PSA-binding signals of the highly metastatic cells compared to the parental cells were also observed in both cell lines (Fig. 3d, e) (*P* < 0.05). This may indicate the involvement of high mannose type N-glycans in the metastatic phenotype of CCA cells.

### Cell surface high mannose type N-glycans promote progression of CCA metastasis

**Fig. 4:**
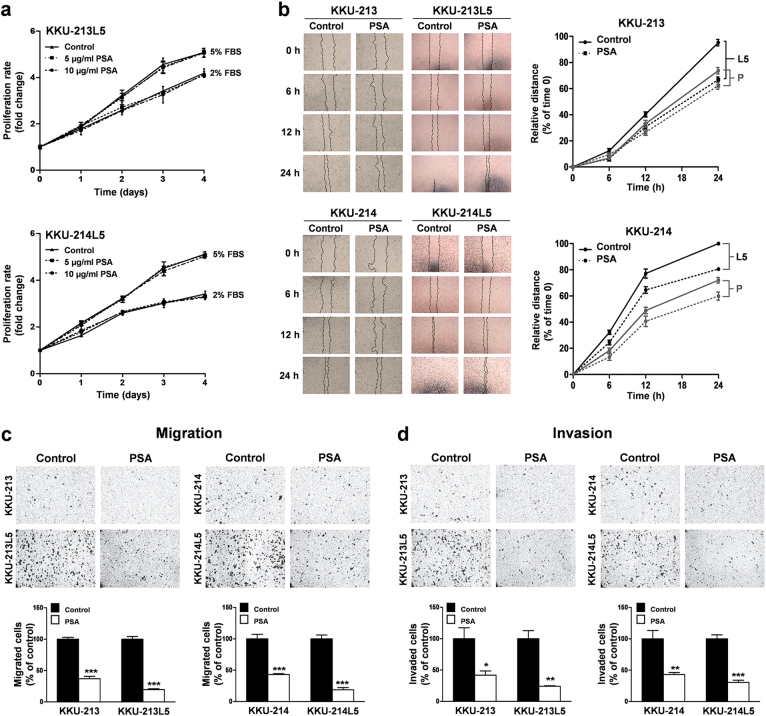
PSA treatment reduced migration and invasion abilities of CCA cells. Treatment of PSA in the parental cells, KKU-213 and KKU-214, and highly metastatic CCA cells, KKU-213L5 and KKU-214L5, for 24 h had no effect on (**a**) cell proliferation, but significantly affected (**b**, **c**) cell migration and (**d**) invasion. The numbers of migrated and invaded cells were compared with the non-treated control cells given as 100%. The results are mean ± SEM of two independent experiments; *P *<* 0.05, **P *<* 0.01, ***P *<* 0.001, student’s *t* test

To demonstrate the association of cell surface high mannose type N-glycans with CCA progression, the surface high mannose type N-glycans were masked with PSA prior to metastatic activity assays. To prevent the possible cell aggregation caused by lectin binding, the appropriate concentration of PSA was first optimized. CCA cells were treated with 0–100 µg/ml of PSA for 1 h and the aggregated cells were determined under a microscope. No aggregated cells were observed at PSA 6.25–12.5 µg/ml (Supplementary Figure S2), therefore, PSA at 5 and 10 µg/ml were used to neutralize high mannose glycans at the cell surface. As shown in Fig. 4a, PSA treatment had no effect on growth of CCA cells either in the presence of 2% or 5% fetal bovine serum (FBS). The highly metastatic cells exhibited higher migration capability than the parental cells as shown in the wound scratch and migration assays (Fig. 4b, c). CCA cells treated with 10 µg/ml of PSA did decrease the cell migration abilities of both parental and highly metastatic cells, but to a lesser extent in the parental cells. In the wound scratch assay, PSA treatment extended the time of wound closing of highly metastatic cells from 12 h to more than 24 h (Fig. 4b). Moreover, treated cells with PSA significantly decreased the migratory abilities of the parental cells to 40% and those of highly metastatic cells to 20% relatively to the control cells (*P* < 0.001) (Fig. 4c). Similar effects were observed in the invasion assay. PSA treatment markedly decreased the number of invaded cells by 42–43% for parental cells (*P* < 0.05; *P* < 0.01) and 25–30% for highly metastatic cells (*P* < 0.01; *P* < 0.001) in both cell lines (Fig. 4d). These data highlight the significance of the surface high mannose type N-glycans in promoting the progression of CCA metastasis.

### O-GlcNAcylation upregulates high mannose type N-glycans via decreasing MAN1A1 and FOXO3 expression

**Fig. 5:**
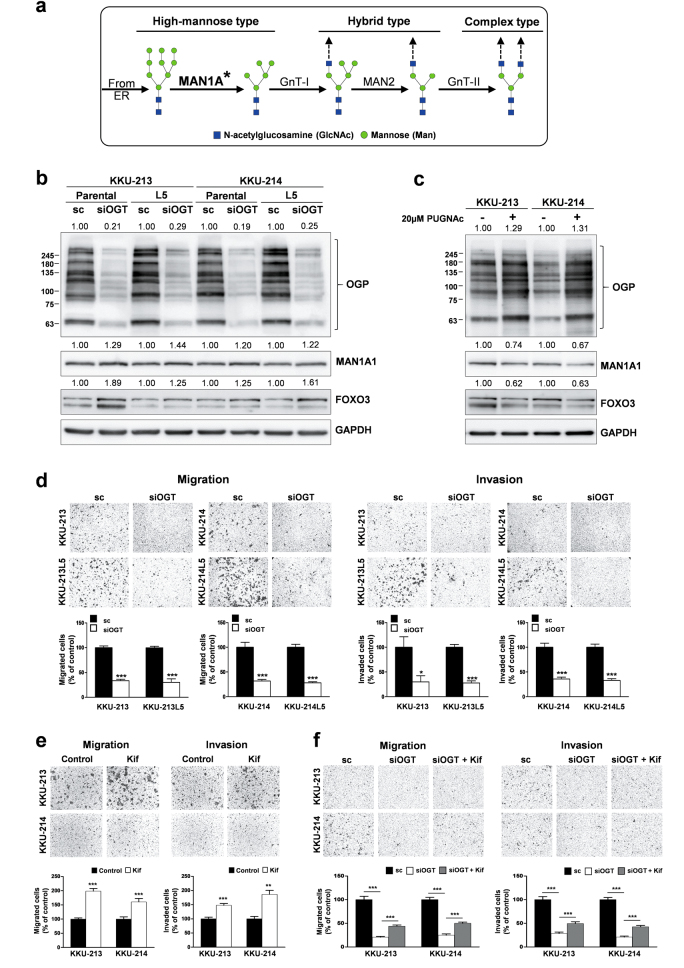
O-GlcNAcylation regulated migration and invasion abilities of CCA cells via FOXO3 and MAN1A1 expression. **a** N-linked glycan synthesis pathway in Golgi apparatus. **b** siOGT effectively suppressed O-GlcNAcylation in parental (KKU-213 and KKU-214) and highly metastatic (KKU-213L5 and KKU-214L5) CCA cells as the levels of O-GlcNAcylated proteins (OGP) were reduced. The siOGT treatment suppressed expression of MAN1A1 and FOXO3. **c** PUGNAc treatment could increase OGP and reduce the expression of MAN1A1 and FOXO3 in the parental cells. GAPDH was used as the loading control, data represent one of two independent experiments. **d** Suppression of O-GlcNAcylation by siOGT reduced migration and invasion abilities of parental and highly metastatic CCA cells. **e** Inhibition of MAN1A1 activity using kifunensine (Kif) enhanced migration and invasion abilities of CCA cells. **f** The inhibition effect of siOGT on CCA migration/invasion could be reversed by Kif treatment. The results are mean ± SEM of one representation from two independent experiments; *P *<* 0.05, **P *<* 0.01, ***P *<* 0.001, student’s *t* test

The molecular mechanisms by which O-GlcNAcylation controlled the expression level of high mannose type N-glycans were further explored. The biosynthesis of all N-glycans starts in the endoplasmic reticulum (ER), and the elongation process for the peripheral glycan moieties occurs in the Golgi apparatus [18, 31]. The core N-glycan structure is mannosylated in the ER to yield Hex_9_HexNAc_2_, trimmed back by α1,2-mannosidases, e.g., α1,2-mannosidase IA (MAN1A1) in the Golgi apparatus to form Hex_5_HexNAc_2_, and further elongated to hybrid and complex types (Fig. 5a). Therefore, the accumulation of Hex_9_HexNAc_2_ is likely due to the decrease in expression of MAN1A1. We first tested whether MAN1A1 expression was modulated via O-GlcNAcylation. The expression of MAN1A1 was determined in the parental and highly metastatic CCA cells treated with siOGT. As shown in Fig. 5b, siOGT significantly reduced the level of O-GlcNAcylation as determined by the levels of O-GlcNAcylated proteins (OGP). Compared to untreated cells, siOGT treatment dramatically reduced O-GlcNAcylation of the parental cells 0.20-fold and of the highly metastatic cells 0.25 fold. Suppression of O-GlcNAcylation indeed increased expression of MAN1A1 in all siOGT-treated cells. In contrast, inhibition of OGA by PUGNAc, elevated O-GlcNAcylation and decreased MAN1A1 expression compared with those of control cells (Fig. 5c). The data supports the link between O-GlcNAcylation levels and MAN1A1 expression.

As FOXO3, a member of Forkhead box (FOX) transcriptional factors, has been suggested to regulate MAN1A1 expression (http://www.sabiosciences.com/chipqpcrsearch.php), we next tested whether FOXO3 expression was also modulated by O-GlcNAcylation. Similar to MAN1A1, the expression of FOXO3 increased in all siOGT-treated cells and decreased in PUGNAc treated cells (Fig. 5b, c). The data imply that O-GlcNAcylation affects MAN1A1 expression by modulating FOXO3 expression.

To reveal whether O-GlcNAcylation affects CCA metastasis, the progression of the parental and highly metastatic CCA cells were compared in siOGT and scramble siRNA-treated cells. siOGT treatment suppressed O-GlcNAcylation and reduced the migration and invasion abilities of both parental and highly metastatic CCA cells (*P* < 0.001) (Fig. 5d). To demonstrate whether MAN1A1 affects migration and invasion of CCA cells, MAN1A1 activity was inhibited using kifunensine, a specific inhibitor of mannosidase 1. As demonstrated in Fig. 5e, the migration/invasion abilities of kifunensine treated cells were significantly increased relatively to those of the control cells. Moreover, the inhibition effect of OGT silencing on the reduction of migration and invasion could be rescued by kifunensine treatment ~2 fold compared to siOGT-treated cells (Fig. 5f). These data support the connection of O-GlcNAc modifcation and MAN1A1 with the migration and invasion capabilities of CCA cells.

### O-GlcNAcylation regulates MAN1A1 expression via activating Akt/Erk signaling and modulating FOXO3 stability

**Fig. 6:**
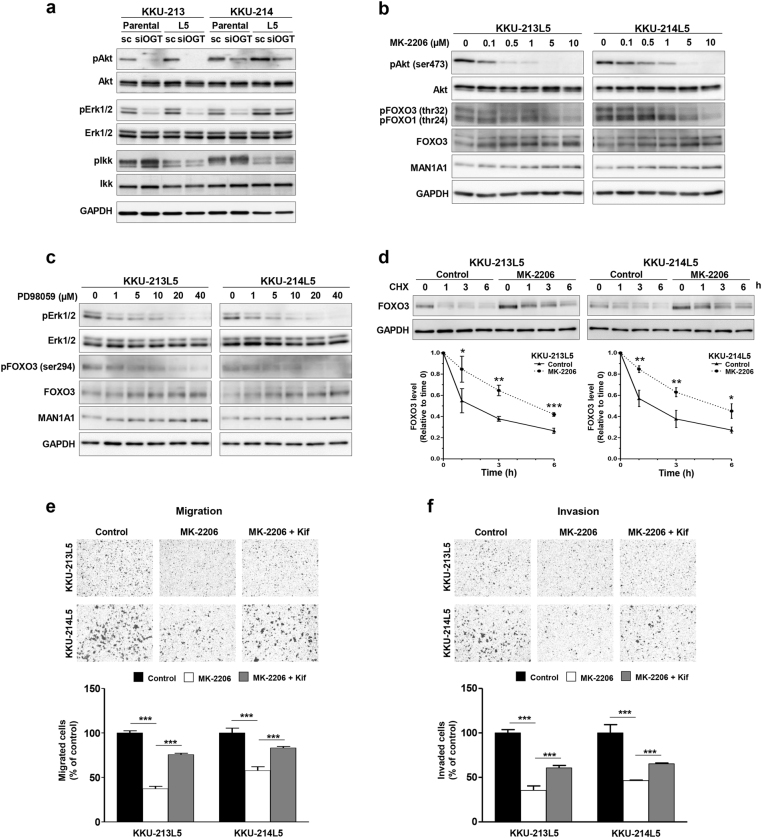
O-GlcNAcylation regulated activations of Akt and Erk and expression of FOXO3 and MAN1A1. OGT was suppressed in parental (KKU-213 and KKU-214) and highly metastatic (KKU-213L5 and KKU-214L5) CCA cells using siOGT. **a** siOGT treatment inactivated the phosphorylation of Akt and Erk but not Ikk. **b** The modulations of Akt and Erk on FOXO3 expression were revealed using Akt and Erk inhibitors. CCA cells were treated with various concentrations of Akt inhibitor, MK-2206 and (**c**) Erk inhibitor, PD98059, for 24 h. The expression of pAkt/Akt, pErk/Erk, phospho-FOXO3, FOXO3 and MAN1A1 were determined using western blotting. **d** The MK-2206-treated L5 CCA cells were incubated with 20 µM of cycloheximide (CHX) for 1, 3, and 6 h. FOXO3 levels at each time point were determined using western blotting and compared with those of the untreated control cells. The data are represented as the mean ± SD from three independent experiments. Inhibition of Akt activation significantly reduced (**e**) migration and (**f**) invasion abilities of CCA cells and these effects could be rescued by kifunensine (Kif) treatment. The results are mean ± SEM of one representative from two independent experiments. *P *<* 0.05; **P *<* 0.01; ***P *<* 0.001, student’s *t* test

We further elucidated how O-GlcNAcylation regulates expression of MAN1A1 via FOXO3. The activations of Akt, Erk, and Ikk, which are modulated by O-GlcNAc modification and are the major kinases that regulate the expression of FOXO3 [32], were proposed to be the connection between O-GlcNAcylation and FOXO3. The phosphorylations of Akt, Erk, and Ikk were hence determined in the siOGT-treated cells. As shown in Fig. 6a, the levels of pAkt/Akt and pErk/Erk, but not pIkk/Ikk, were markedly decreased in the OGT knocked down cells. All the data at this stage indicated that suppression of O-GlcNAcylation inhibits the activation of Akt and Erk, which subsequently upregulates FOXO3 activity and MAN1A1.

We next explored whether activation of Akt and Erk directly modulates FOXO3 expression. An allosteric inhibitor of Akt, MK-2206, was used to inhibit Akt activity in the high metastatic CCA cells. In the presence of MK-2206, phosphorylation of Akt was reduced in a dose dependent manner. As a result, phosphorylation of FOXO3 at Thr-32 was suppressed, whereas expression of total FOXO3 and MAN1A1 were dependently increased with increasing doses of MK-2206 (Fig. 6b). Similar observations were obtained when PD98059 (2′-amino-3′-methoxyflavone), a selective MEK inhibitor, was used to inhibit Erk activation. Phosphorylation of Erk was reduced by PD98059 treatment in a dose dependent fashion. The treatment also decreased phosphorylation of FOXO3 at Ser-294 and increased expression of FOXO3 and MAN1A1 (Fig. 6c). The inactivation of Akt and Erk signaling pathways had no effect on the status of O-GlcNAcylation in CCA cells (Figure S3). These results demonstrate the direct effects of Akt and Erk activation on FOXO3 and MAN1A1 expression.

As Akt phosphorylation of FOXO3 has been shown to increase the degradation of FOXO3, we next explored whether Akt affects FOXO3 stability in CCA cells. The stability of FOXO3 was determined following cycloheximide (CHX) treatment. As shown in Fig. 6d, the stability of FOXO3 was prolonged by MK-2206 treatment, with a half-life of ∼5 h for MK-2206 treated cells and ∼1.5 h for the control cells. Together, Akt/Erk inhibitors inactivated Akt/Erk phosphorylation, resulting in the reduction of FOXO3 phosphorylation, which in turn increased the stability of FOXO3 and expression of MAN1A1.

To demonstrate the link between Akt activation and the function of MAN1A1 on CCA progression, we first investigated the migration and invasion of CCA cells in the presence of an Akt inhibitor, MK-2206. As shown in Fig. 6e, MK-2206 treatment markedly decreased the migration of CCA to 40% and 60% in KKU-213L5 and KKU-214L5, respectively. Moreover, these effects could be reversed by inhibiting MAN1A1 activity using kifunensine. Treatment of cells with MK-2206 and kifunensine significantly increased the motility 1.4–2 folds higher than those of MK-2206 treated cells (*P* < 0.001). Similar results were observed for their invasion abilities (Fig. 6f). Collectively, our data show that O-GlcNAcylation promotes progression of CCA metastasis via activation of Akt, which consequently represses FOXO3 and MAN1A1 expression.

### FOXO3 positively regulates MAN1A1 expression in CCA cells

**Fig. 7:**
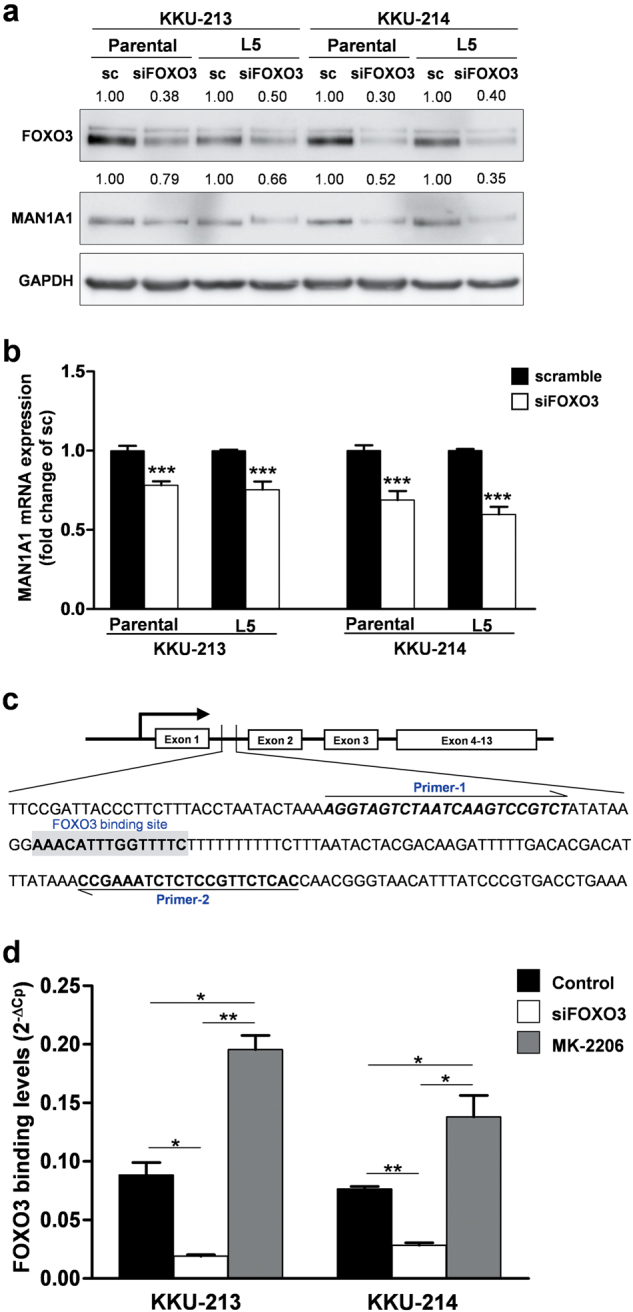
FOXO3 is a positive regulator of MAN1A1. CCA cells were treated with siFOXO3 for 48 h. The expression of FOXO3 and MAN1A1 were determined using western blots and real-time PCR. Compared with scramble (sc) treated cells, suppression of FOXO3 using siFOXO3 decreased expression of FOXO3 and expression levels of (**a**) MAN1A1 protein and (**b**) MAN1A1 mRNA. GAPDH was used as the loading control. **c** Schematic illustrations of the locations of primers and FOXO3-binding region on the *MAN1A1* gene used in the ChIP assays. **d** Chromatin immunoprecipitation (ChIP) assay was used to determine the direct binding of FOXO3 on *MAN1A1* gene. To confirm the binding activity of FOXO3 on *MAN1A1* gene, ChIP assays of cells treated with siFOXO3 and MK-2206 for 24 h were performed and compared to those of control cells. The results are one representation from two independent experiments. *P < 0.05, **P < 0.01, ***P *<* 0.001, student’s *t* test

As the level of MAN1A1 expression corresponded well with FOXO3 expression, we further questioned whether FOXO3 is a direct modulator of MAN1A1 expression. We first verified the association of FOXO3 and MAN1A1 by determination of MAN1A1 expression in siFOXO3 treated cells. siFOXO3 treatment effectively suppressed FOXO3 and MAN1A1 expression in both parental and highly metastatic CCA cells (Fig. 7a). Suppression of FOXO3 expression could also significantly suppress expression levels of MAN1A1 mRNA and protein (*P* < 0.001, Fig. 7b).

To prove the direct regulation of FOXO3 on MAN1A1 expression, chromatin immunoprecipitation (ChIP) assay was performed. The primer sequences were designed to cover the binding region of FOXO3 on *MAN1A1* gene as revealed by EpiTect ChIP qPCR Primers (Fig. 7c). ChIP assay was conducted in the parental cells, in the presence and absence of siFOXO3 and MK-2206. The positive binding of FOXO3 on *MAN1A1* gene was detected, as shown in Fig. 7d and Aupplementary Figure S4. Moreover, suppression of FOXO3 by siFOXO3 reduced the FOXO3-binding level, whereas increase of FOXO3 by inhibiting Akt activity using MK-2206 significantly enhanced the FOXO3 binding on the endogenous *MAN1A1* gene in both KKU-213 and KKU-214 cell lines. Taken together, this information suggests for the first time that FOXO3 is a positive direct regulator of MAN1A1.

### Association of O-GlcNAcylation, high-mannose type N-glycans and MAN1A1 observed in the in vitro studies were confirmed in patients’ tissues

**Fig. 8:**
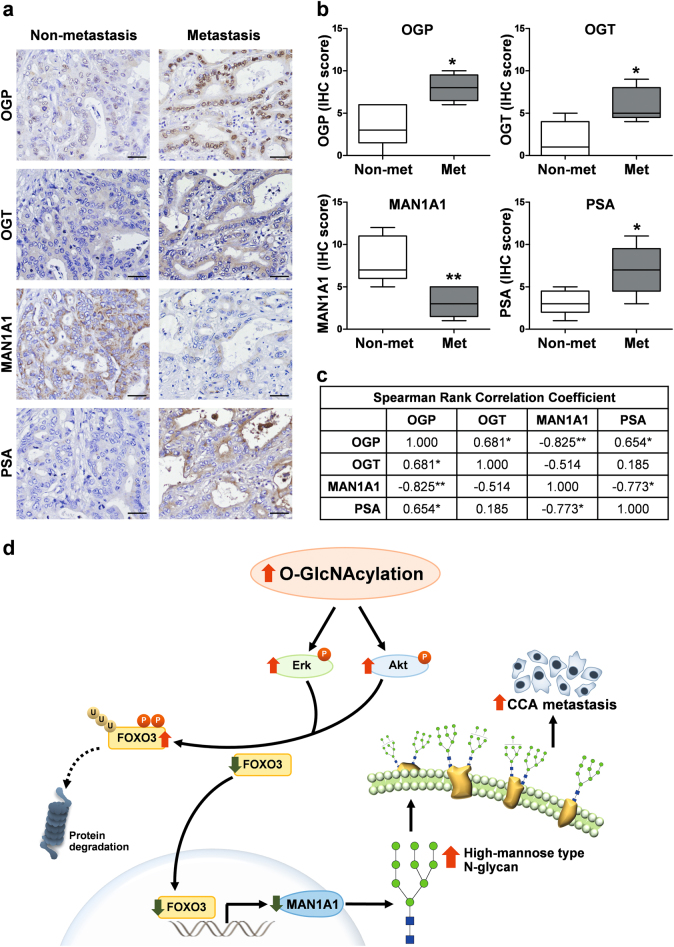
CCA tissues with metastasis exhibit high expression of OGP, OGT, and strong PSA binding, with low MAN1A1. **a** The expression of OGP, OGT, MAN1A1 and PSA were determined using IHC staining non-metastatic (*n* = 5) and metastatic (*n* = 5) CCA tissues. Bars indicate 20 µm. **b** Mean expression of OGP, OGT and PSA in metastatic CCA (Met) are significantly higher than that in non-metastatic CCA (Non-met). The opposite was observed for MAN1A1 expression (*P < 0.05, **P < 0.01; Mann–Whitney test). **c** The correlations between OGP, OGT, MAN1A1 and PSA levels were shown by Spearman’s rank correlation test (*P < 0.05, **P < 0.01). **d** Schematic diagram demonstrates the mechanism of O-GlcNAcylation augmenting metastasis of CCA cells via increasing high mannose type N-glycans. Elevation of O-GlcNAcylation activates Akt and Erk activities, which further induces phosphorylation of FOXO3. The phosphorylation of FOXO3 by Akt or Erk leads to proteolytic degradation of FOXO3. The decrease of FOXO3 may cause the reduction of MAN1A1 expression, leading to the elevation of high mannose type N-glycans on the cell surface. The increase of the high mannose type N-glycan, Man 9, can promote CCA metastasis

Upon demonstrating the correlation of OGP, OGT, MAN1A1 and PSA with cell migration/invasion of CCA cell lines (Figs 3–5), we next explored whether the same could be observed in CCA tissues of patients. Immunohistochemistry of OGP, OGT, MAN1A1 and PSA-histochemistry were performed in five each of non-metastasis and metastasis CCA cases. As shown in Fig. 8a, b, the expression of OGP, OGT and PSA were elevated in CCA tissues with metastasis compared to those with non-metastatic CCA. In contrast, MAN1A1 expression in CCA tissues with metastasis was lower than that in non-metastatic CCA. The correlation of OGP, OGT, MAN1A1 and PSA levels in CCA tissues were determined using Spearman rank correlation coefficient. As shown in Fig. 8c, OGP had a positive correlation with expression of OGT and PSA but had a negative correlation with expression of MAN1A1. In addition, OGT expression was correlated with OGP levels while PSA signal was negatively correlated with MAN1A1 expression. These in vivo data confirmed the associations of O-GlcNAcylation, high-mannose type N-glycan and MAN1A1 observed in the in vitro studies.

## Discussion

The glycan moieties of glycoproteins and glycolipids on cell membrane play several important biological roles. Incomplete synthesis and neo-synthesis of glycans are commonly found in cancer cells [33]. The regulation of glycan synthesis, however, is not well understood. The rationale of this study for the contribution of O-GlcNAcylation on regulation of N-glycans was initiated from several observations. First, elevation of O-GlcNAcylation enhances CCA progression [25, 26]. Second, increase of several membranous N-glycans are related to progression of cancer [34–36]. Finally, O-GlcNAcylation can regulate many transcriptional factors that modulate the expression of several proteins including enzymes. These lines of evidence led us to hypothesize that O-GlcNAcylation may enhance progression of CCA by modulation of membranous N-glycans via glycan synthesizing enzymes.

Our study is the first report to demonstrate that O-GlcNAcylation, an intracellular glycosylation, can modulate the expression of a cell surface high mannose type N-glycan, Hex_9_HexNAc_2_, and consequently promote progression of CCA cells. The molecular link between O-GlcNAcylation and high mannose type N-glycans via activation of Akt/Erk, and expression of FOXO3 and MAN1A1 was elucidated. Moreover, the direct regulation of MAN1A1 expression by FOXO3 was first evidenced in this study.

Overexpression of OGT in tumor tissues and the association between high O-GlcNAcylation and poor patients’ outcome have been previously reported in CCA [24]. The in vitro study indicated that the abilities of CCA cells to migrate and invade could be monitored by modulating O-GlcNAcylation levels [25]. In addition, higher level of O-GlcNAc modification in highly metastatic CCA cells than the parental cells were hitherto observed [26]. In the present study, we further demonstrate the molecular pathway by which O-GlcNAcylation modulates progression of CCA. As the glycan moieties of membrane proteins have a significant function in adhesion of cell–cell/cell–matrix, cell invasion and migration, we postulated that O-GlcNAcylation modulates specific glycan moieties of membrane proteins. Comparing the glycomic data of membranous N-glycans between low and highly metastatic CCA cells and between siOGT treated and scramble control cells suggested a membranous high mannose type N-glycan, Hex_9_HexNAc_2_, and a biantennary complex type N-glycan, Hex_5_HexNAc_4_Fuc_1_NeuAc_1_, to be associated with metastatic activity and be mediated by O-GlcNAcylation. Up to our search, this study is the primary report of the link between O-GlcNAcylation and the expression of high mannose type N-glycans.

Normally, mammalian cells scarcely express cell surface high mannose type N-glycans [37], with the exemption of stem cells and macrophages [38]. In the current study, these N-glycans were the highest abundant N-glycans found on the membranous glycoproteins of CCA cells, indicating dynamic changes of glycosylation in CCA progression. In the current study, the significant increase of Hex_9_HexNAc_2_ level in the highly metastatic cells was evident from the glycomic analysis data (Fig. 2c) and PSA-cytofluorescence signals (Fig. 3e). The involvement of high mannose type N-glycans and progression of CCA cells was confirmed by the PSA neutralizing experiment. Masking high mannose type N-glycans with PSA, a mannose binding lectin, significantly reduced the capabilities of CCA cells in migration and invasion. Notably, the effect of PSA inhibition on migration and invasion was more obvious in highly metastatic cells than in the parental cells. This implies that the magnitude of high mannose type N-glycans is positively related to the progression of CCA metastasis. The association of overexpression of Hex_9_HexNAc_2_ with metastasis has been reported in several types of cancers [34–36]. In addition, Hex_8_HexNAc_2_ glycan was also stated in pancreatic tumor tissues [39].

O-GlcNAcylation, an intracellular glycosylation, is involved in key cellular processes, including nutrient sensing, stress response, transcription-translation, signal transduction, and proteolysis by proteasome [21]. To our knowledge, the connection of O-GlcNAc modification and the Hex_9_HexNAc_2_ expression has never been reported. We demonstrate that reduction of O-GlcNAcylation with siOGT significantly reduced cell surface high mannose type N-glycans. To elucidate how O-GlcNAcylation modulates the level of Hex_9_HexNAc_2_, we first focused on the expression of MAN1A1, as it is the enzyme that mediates Hex_9_HexNAc_2_ processing by removing 3 terminal mannose residues from Hex_9_HexNAc_2_. Suppression of O-GlcNAcylation using siOGT elevated the expression level of MAN1A1 and consequently reduced the level of Hex_9_HexNAc_2_. Conversely, induction of O-GlcNAcylation using an OGA inhibitor, PUGNAc, decreased the expression of MAN1A1. These data support our hypothesis that O-GlcNAcylation can regulate the level of surface N-glycan via modulating the expression of glycan synthesizing enzymes, namely MAN1A1. The significance of O-GlcNAcylation and MAN1A1 on the progression of CCA metastasis was confirmed by the facts that reduction of O-GlcNAcylation markedly suppressed the migratory and invasion abilities of both parental and highly metastatic CCA cells (Fig. 5d), whereas inhibition of MAN1A1 activity by kifunensine increased the observed functions (Fig. 5e). We further show O-GlcNAcylation modulates the progression of CCA metastasis via MAN1A1 by the finding that reduction of CCA metastasis by siOGT treatment could be reversed by inhibiting MAN1A1 activity using kifunensine (Fig. 5f).

We next explored how O-GlcNAcylation regulates MAN1A1 expression. Our previous study demonstrated that O-GlcNAcylation could activate Akt signaling and promote progression of CCA metastasis [25]. This information supports our finding that FOXO3, a transcriptional factor under Akt and Erk activation, is a transcriptional factor of MAN1A1. Our data led us to propose that FOXO3 is a transcriptional factor that linked O-GlcNAcylation and Akt and Erk activation to MAN1A1 expression. We demonstrate the link between FOXO3 and expression of MAN1A1 by the findings that suppression of FOXO3 expression decreased the expression level of MAN1A1 in both parental and highly metastatic cells. In addition, the analysis of EpiTect ChIP qPCR Primers assay suggested FOXO3 as a possible transcriptional regulator of *MAN1A1*. The ChIP assay showed that FOXO3 could bind to the *MAN1A1* gene and that DNA binding of FOXO3 correlated well with the expression levels of FOXO3, especially in the FOXO3 suppressed cells or Akt inhibited cells. These data demonstrated for the first time that FOXO3 is a positive transcriptional activator of MAN1A1.

The association of O-GlcNAcylation and FOXO3 expression was further shown to be via Akt and Erk activation. First, suppression of O-GlcNAcylation using siOGT was able to inhibit the activation of Akt and Erk in both parental and highly metastatic CCA cells. Second, inhibiting activities of Akt and Erk by their specific inhibitors reduced the phosphorylation of FOXO3 at Thr-32 and Ser-294, leading to the increased expression of FOXO3 and hence MAN1A1.

In agreement with our findings, the association of O-GlcNAc modification and increased metastatic ability has been shown in several types of cancers. Activation of PI3K/Akt and MAPK/Erk pathways have been demonstrated to be the link between O-GlcNAcylation and metastatic ability of various cancer cells, including thyroid anaplastic cancer [40] and breast cancer [41]. It has been shown that elevation of O-GlcNAcylation by OGA inhibition or overexpression of OGT could increase Akt phosphorylation at Ser-473 in thyroid anaplastic cancer cells [40]. In addition, suppression of O-GlcNAcylation by siOGT decreased O-GlcNAcylation and phosphorylation of Akt in breast cancer cells [41]. In primary mouse vascular smooth muscle cells, O-GlcNAcylation of Akt at Thr-430 and Thr-479 promoted Akt phosphorylation at Ser-473 and consequently induced vascular calcification [42].

Furthermore, similar to our finding, earlier reports suggest that activation of Akt and Erk promotes cancer progression via downregulation of FOXO3 [32] in breast cancer [43] and uveal melanoma cancer cells [44]. The mechanism by which Erk activation promotes breast cancer progression via inhibition of FOXO3 expression has also been shown to be through MDM2-mediated degradation [45]. This evidence is consistent with our study that suppression of Akt using the protein synthesis inhibitor cycloheximide increases the expression level of FOXO3 by stabilizing the protein. The connection between Akt activation and MAN1A1 on CCA progression has also been demonstrated for the first time in this study. Inhibition of Akt activity markedly inhibited migration and invasion of highly metastatic CCA cells and these effects could be rescued by inhibiting MAN1A1 activity using kifunensine (Fig. 6e, f).

The correlation between MAN1A1 expression and metastasis has been reported in liver cancer. Lower expression of MAN1A1 in HCCLM3, MHCC97H, and MHCC97L, the metastatic hepatoma cells than Hep3B, the non-metastatic hepatoma cells was reported [46]. Moreover, high expression of MAN1A1 was correlated with a better outcome of breast cancer patients [47]. The regulation of MAN1A1 in cancer, however, is still unclear. It has been shown in melanoma, liver, breast and cervical cancers that aberrant expression of MAN1A1 is associated with the status of methylation at promoter region [48]. Expression level of MAN1A1 was shown to be modulated via FOXO3 in the present study. MAN1A1 and FOXO3 expression in CCA tissues and their association with clinical pathological features of the patients should be explored to support the in vitro findings of this study. Moreover, further investigation is needed on the roles of the biantennary structure, Hex_5_HexNAc_4_Fuc_1_NeuAc_1_, as well as the truncated complex N-glycans, Hex_5_HexNAc_3_Fuc_1_ and Hex_5_HexNAc_4_Fuc_1_, in O-GlcNAc-mediated CCA progression.

Our earlier studies demonstrated the increase of O-GlcNAcylation in CCA tissues with negative relationship to survival of CCA patients [24]. In addition, elevation of O-GlcNAcylation has been shown to increase progressiveness of CCA metastasis via activation of Akt and Erk [25]. In the current study, the molecular link between O-GlcNAcylation and CCA progression was further shown to be via increasing of the membranous high mannose type N-glycan, Hex_9_HexNAc_2_. O-GlcNAcylation activates the PI3K/Akt and MAPK/Erk signaling pathways which negatively regulates the expression level of FOXO3 leading to the decreased expression of MAN1A1 and accumulation of Hex_9_HexNAc_2_ (Fig. 8d). These findings reveal new perspectives of Hex_9_HexNAc_2_ as a candidate marker and therapeutic target for CCA.

## Materials and methods

### Antibodies and reagents

Antibodies were obtained from several sources: anti-O-GlcNAc (RL-2, #MA1-072) from Pierce Biotechnology (Rockford, IL); anti-MAN1A1 (#M-3694) from Sigma-Aldrich (St. Louis, MO); anti-pAkt (ser-473, #9271s), anti-Akt, anti-pFOXO3 (ser-294, #5538s), and anti-pFOXO3 (thr-32, #9464s) from Cell Signaling (Danvers, MA); anti-Erk (K-23, #sc-94), anti-pErk (E-4, #sc-7383), anti-pIkk (T23, #sc-101706), and anti-Ikk (H470, #sc-7607) from Santa Cruz Biotechnology; anti-FOXO3 (#07–702) and anti-GAPDH (#MAB-374) from Merck Millipore (Billerica, MA). Akt inhibitor, MK-2206 dihydrochloride (#sc-364537) was from Santa Cruz Biotechnology (Santa Cruz, CA) and Erk inhibitor, PD98059 (#9900) was obtained from Cell Signaling (Danvers, MA). PNGase-F (#P0704) was purchased from New England Biolabs (Ipswich, MA), *Pisum Sativum* Agglutinin (PSA, #L-1050), biotinylated PSA (#B-1055), biotinylated Concanavalin A (#B-1005) were obtained from Vector Laboratories (Burlingame, CA). Mannosidase I inhibitor, kifunensine (#K1140), OGA inhibitor, [O-(2-Acetamido-2-deoxy-D-glucopyranosylidenamino)-N-phenylcarbamate; PUGNAc, #A7229], and cycloheximide (CHX, #C104450) were purchased from Sigma-Aldrich.

### Cell culture and treatments

KKU-213 and KKU-214, the CCA cell lines were provided from the Japanese Collection of Research Bioresources (JCRB) Cell Bank, Osaka, Japan. Highly metastatic CCA sublines; KKU-213L5 and KKU-214L5 were established from the parental cells, KKU-213 [49] and KKU-214 [50]. All cells were cultured in HAM’s F-12 (Gibco, NY) containing 10% fetal bovine serum (Gibco) and 1% antibiotic-antimycotic (Gibco), under the standard protocol at 37 °C and 5% CO_2_.

OGA inhibitor, PUGNAc, was used to enhance O-GlcNAcylation level in CCA cells. CCA cells, (8 × 10^5^ cells) in a 6 cm-culture dish were treated with 20 µM PUGNAc for 24 h prior to further experiments.

Cycloheximide (CHX) a protein synthesis inhibitor, was used to determine protein stability. Adherent CCA cells (3 × 10^5^ cells/well) in a 6-well plate were treated with 20 µg/ml of CHX for the indicated time.

Kifunensine, a mannosidase 1 inhibitor, was used to inhibit the MAN1A1 activity. After plating 8 × 10^5^ cells per well in a 6 cm-culture dish for 24 h, 20 µg/ml of kifunensine was added in each well and the plates were cultured further for 48 h before subjecting to further experiments.

PSA neutralization test was used to neutralize cell surface mannosylated N-glycans. CCA cells (3 × 10^5^ cells) were treated with 10 µg/ml of PSA in a complete media for 24 h at 37 ^o^C prior to further experiments.

### OGT- and FOXO3- siRNA-treated cells

The level of O-GlcNAcylation was modulated by suppressing OGT expression using specific siRNA as described previously [51]. The negative control siRNA (scramble siRNA, QIAGEN, Hilden, Germany) was used as the control. Similar procedures were performed for siFOXO3 [52].

### Preparation of membrane fraction and purification of membranous N-glycans

The membrane fraction was harvested according to the standard procedure [53] with minor modification. In brief, CCA cells were homogenized in 20 mM HEPES-KOH, pH 7.4 containing 0.25 M sucrose and 1:100 protease inhibitor, using probe sonication (25 Amplitude, pulsed on 5 s and off 10 s for 5 times). Cell nuclei were fractionated by centrifugation at 2000 × *g*, for 10 min. Then, the membrane fraction was separated by 3 times of ultra-centrifugation at 200,000 × *g*, 45 min. The final pellet was collected as the membrane fraction and total N-glycans were released using 1000 U of PNGase-F at 37 °C in a microwave reactor (CEM Corporation, Matthews, NC) at 20 watts for 10 min. The released N-glycans were enriched and purified by porous graphitized carbon solid-phase extraction as described previously [48]. The unbound fraction was first eluted with 9 ml of pure water and the bound N-glycan fraction was eluted with 4 ml of 40% ACN and 0.1% TFA in pure water.

### Glycomic analysis of membranous N-glycans

Samples were reconsitituted in 30 µl pure water and composition of glycans were analyzed by Agilent HPLC-Chip/Time-of-Flight (Chip/TOF) MS system (Agilent Technologies, Santa Clara, CA) as previously reported [48]. The enrichment column was conditioned with 3% ACN and 0.1% formic acid (FA) in water. Sample (5 μl) was injected at 4.0 μl/min using a 6 °C maintained autosampler. The mass spectra were analyzed using Agilent MassHunter Qualitative Analysis software and the extracted compound chromatograms (ECC) were determined using the Molecular Feature Extractor algorithm. Each compound was identified using an in-house retrosynthetic library [54]. Area under the peaks was used for relative quantitation.

### Cell aggregation test

To select the appropriated concentration of *Pisum Sativum* Agglutinin (PSA), a mannose recognition lectin, cell aggregation test was first tested. In a 24-well plate, KKU-213L5 of 5 × 10^4^ cells per well were incubated in a serum free Ham’s F-21 media containing 0–100 µg/ml of PSA for 1 h. The aggregated cells were visualized using a light microscope. The highest PSA concentration that did not initiate aggregated cells was selected for further studies.

### PSA-cytofluorescence staining

A solution of 4% paraformaldehyde was used to fix the cells for 30 min prior to block the non-specific binding with 0.5% BSA in PBS for 5 min. Cells were then incubated with 0.01 mg/ml of biotinylated PSA (Vector Laboratories) at 4 °C, overnight and 1 h at room temperature with 1:500 streptavidin-FITC (Invitrogen, UK). Hoechst 33342 (Molecular probe, Invitrogen) was used to stain the nuclei and the fluorescent image was visualized using a ZEISS LSM 800 Confocal Laser Scanning Microscope (Zeiss, Oberkochen, Germany). The intensities of the fluorescent signals were analyzed using ZEN 2.1 software (Zeiss).

### Cell proliferation

Viable cells in a 96-well plate were determined continuously for 4 days using the MTT proliferation assay (Moleular probes, Eugene, OR) as previously described [25]. Proliferation rate (fold change from control) was determined as OD of treatment/mean OD of control.

### Cell migration and invasion

The Boyden chamber assay (8.0 µm pore size, Corning Incorporated, Corning, NY) was used to determine the migration and invasion as previously described [25]. For invasion assay, the upper chambers were pre-coated overnight with 100 µl of 0.4 mg/ml Matrigel (BD Matrigel^TM^, BD biosciences). At least 5 microscopic fields of 10 × objective were determined for migrated and invaded cells. Triplicate tests per experiment were performed.

### Western blot analysis

Cell lysate (30–50 µg) in a NP-40 lysis buffer was loaded onto a 10% SDS-PAGE and the separated proteins were electro-transferred onto a PVDF membrane. The immunoreactivity and captured signal were determined as mentioned previously [25]. Amount of proteins in cell lysates were measured with Bradford reagent (Bio-rad laboratories, Hercules, CA) as described by the manufacturer.

### RNA extraction and real-time reverse transcriptase polymerase chain reaction (RT-PCR)

Total RNA extraction and cDNA conversion were performed as described previously [25]. PCR reactions were performed using 40 ng cDNA, 0.4 µM of the specific forward and reverse primers for MAN1A1 (5′-GTGGACAGTGGGGTCAACAT-3′ and 5′- GCTGCTAGACTTGCGGATCA-3′) in a total of 10 µl LightCycle 480^®^ SYBR green I master mix (Roche Diagnostic, Mannheim, Germany), using the LightCycle 480^®^ real-time PCR system (Roche Diagnostic). β-actin expression was analyzed as an internal control. Gene expression levels were determined using LightCycle 480^®^ Relative Quantification software (Roche Diagnostic).

### Chromatin immunoprecipitation (ChIP) assay

ChIP assay was performed using the EZ-ChIP^TM^ chromatin immunoprecipitation kit (17–371, Upstate, Millipore Corporation) as suggested by the manufacturer. Briefly, after treating cells with siFOXO3 or Akt inhibitor, the DNA and proteins were crosslinked using formaldehyde at a final concentration of 1% (v/v) in culture media. The cells were sonicated on ice with 5 sets of 15 s pulses on and 10 s pulses off using Ultrasonic homogenizer (sonic VCX 750, Sonics & Materials inc., CT), equipped with a 2 mm tip and set to 30% power. The FOXO3-bound DNA was precipitated with 3 µg/ml of anti-FOXO3 antibody. Rabbit IgG was used as an isotype control. The chromatin-antibody complexes were captured with Protein G-Agarose containing Salmon Sperm DNA. After washing, the DNA–protein cross-links that bound to Protein G-Agarose were eluted using elution buffer, and the complexes were reversed at 65 °C overnight in 0.2 M NaCl. Protein was removed from the DNA using proteinase K at 45 °C for 1 h. The DNA was purified using a spin column. The FOXO3-binding site on *MAN1A1* gene in the purified DNA and input genomic DNA were analyzed by real-time PCR, using the primer sequences; primer-1, 5′-TCCATCAGATTAGTTCAGGCAGA-3′ and primer-2, 5′-CACTCTTGCCTCTCTAAAGCC-3′ as suggested by EpiTect ChIP qPCR Primers (http://sabiosciences.com/chipqpcrsearch.php). The results of FOXO3 binding are normalized against the input DNA.

### Immunohistochemistry

The immunohistochemistry (IHC) experiments were performed using formalin-fixed paraffin-embed liver tissues from histologically proven CCA patients which were obtained from the specimen bank of Cholangiocarcinoma Research Institute at Khon Kaen University (Khon Kaen, Thailand). Each subject gave informed consent and the study protocol was certified by the Ethics Committee for Human Research at Khon Kaen University (HE581369).

Expression levels of OGP, OGT, PSA and MAN1A1 in 10 CCA tissues (non-metastasis (*n* = 5) and metastasis (*n* = 5) CCA) were determined using IHC staining according to the standard protocol [26]. The signals were amplified using the EnVision-system-HRP (Dako, Glostrup, Denmark). The immunoreactivity signals were developed using diaminobenzidine (Sigma-Aldrich). The IHC score was determined as described previously. Two independent assessors scored the levels of IHC staining signal blindly without prior knowledge of clinical parameters.

### Statistical analysis

At least two independent experiments and with two biological replicates were performed in all experiments. GraphPad Prism^®^ 5.0 software (GraphPad software, Inc., La Jolla, CA) was used for statistical analysis. *P* < 0.05 was considered as statistical significance.

## Electronic supplementary material

Supplementary Figures

Supplementary Table S1

Supplementary Table S2

## References

[1] Steeg PS (2016). Targeting metastasis. Nat Rev Cancer.

[2] Sripa B, Pairojkul C (2008). Cholangiocarcinoma: lessons from Thailand. Curr Opin Gastroenterol.

[3] Patel T (2006). Cholangiocarcinoma. Nat Clin Pract Gastroenterol Hepatol.

[4] Uttaravichien T, Bhudhisawasdi V, Pairojkul C, Pugkhem A (1999). Intrahepatic cholangiocarcinoma in Thailand. J Hepatobiliary Pancreat Surg.

[5] Li X, Wang X, Tan Z, Chen S, Guan F (2016). Role of glycans in cancer cells undergoing epithelial-mesenchymal transition. Front Oncol.

[6] Varki A, Freeze HH. Glycans in acquired human diseases. In: Varki A, Cummings RD, Esko JD, Freeze HH, Stanley P, Bertozzi CR, et al, editors. Essentials of glycobiology. 2nd ed. NY: Cold Spring Harbor; 2009; p. 601–16. 20301266

[7] Varki A, Kannagi R, Toole BP. Glycosylation changes in cancer. In: Varki A, Cummings RD, Esko JD, Freeze HH, Stanley P, Bertozzi CR, et al, editos. Essentials of glycobiology. 2nd ed. NY: Cold Spring Harbor; 2009; p. 617–32. 20301279

[8] Christie DR, Shaikh FM, Lucas JAt, Lucas JA, Bellis SL (2008). ST6Gal-I expression in ovarian cancer cells promotes an invasive phenotype by altering integrin glycosylation and function. J Ovarian Res.

[9] Handerson T, Camp R, Harigopal M, Rimm D, Pawelek J (2005). Beta1,6-branched oligosaccharides are increased in lymph node metastases and predict poor outcome in breast carcinoma. Clin Cancer Res.

[10] Litynska A, Przybylo M, Pochec E, Kremser E, Hoja-Lukowicz D, Sulowska U (2006). Does glycosylation of melanoma cells influence their interactions with fibronectin?. Biochimie.

[11] Takahashi M, Kuroki Y, Ohtsubo K, Taniguchi N (2009). Core fucose and bisecting GlcNAc, the direct modifiers of the N-glycan core: their functions and target proteins. Carbohydr Res.

[12] Silsirivanit A, Araki N, Wongkham C, Pairojkul C, Narimatsu Y, Kuwahara K (2011). A novel serum carbohydrate marker on mucin 5AC: values for diagnostic and prognostic indicators for cholangiocarcinoma. Cancer.

[13] Matsuda A, Kuno A, Nakagawa T, Ikehara Y, Irimura T, Yamamoto M (2015). Lectin Microarray-based sero-biomarker verification targeting aberrant O-linked glycosylation on mucin 1. Anal Chem.

[14] Higashi M, Yonezawa S, Ho JJ, Tanaka S, Irimura T, Kim YS (1999). Expression of MUC1 and MUC2 mucin antigens in intrahepatic bile duct tumors: its relationship with a new morphological classification of cholangiocarcinoma. Hepatology.

[15] Tolek A, Wongkham C, Proungvitaya S, Silsirivanit A, Roytrakul S, Khuntikeo N (2012). Serum alpha1beta-glycoprotein and afamin ratio as potential diagnostic and prognostic markers in cholangiocarcinoma. Exp Biol Med.

[16] Indramanee S, Silsirivanit A, Pairojkul C, Wongkham C, Wongkham S (2012). Aberrant glycosylation in cholangiocarcinoma demonstrated by lectin-histochemistry. Asian Pac J Cancer Prev.

[17] Anderson CD, Pinson CW, Berlin J, Chari RS (2004). Diagnosis and treatment of cholangiocarcinoma. Oncologist.

[18] Ghazarian H, Idoni B, Oppenheimer SB (2011). A glycobiology review: carbohydrates, lectins and implications in cancer therapeutics. Acta Histochem.

[19] Hart GW, Housley MP, Slawson C (2007). Cycling of O-linked beta-N-acetylglucosamine on nucleocytoplasmic proteins. Nature.

[20] Zachara NE, Hart GW (2006). Cell signaling, the essential role of O-GlcNAc!. Biochim Biophys Acta.

[21] Butkinaree C, Park K, Hart GW (2010). O-linked beta-N-acetylglucosamine (O-GlcNAc): Extensive crosstalk with phosphorylation to regulate signaling and transcription in response to nutrients and stress. Biochim Biophys Acta.

[22] Hart GW, Slawson C, Ramirez-Correa G, Lagerlof O (2011). Cross talk between O-GlcNAcylation and phosphorylation: roles in signaling, transcription, and chronic disease. Annu Rev Biochem.

[23] de Queiroz RM, Carvalho E, Dias WB (2014). O-GlcNAcylation: the sweet side of the cancer. Front Oncol.

[24] Phoomak C, Silsirivanit A, Wongkham C, Sripa B, Puapairoj A, Wongkham S (2012). Overexpression of O-GlcNAc-transferase associates with aggressiveness of mass-forming cholangiocarcinoma. Asian Pac J Cancer Prev.

[25] Phoomak C, Vaeteewoottacharn K, Sawanyawisuth K, Seubwai W, Wongkham C, Silsirivanit A (2016). Mechanistic insights of O-GlcNAcylation that promote progression of cholangiocarcinoma cells via nuclear translocation of NF-kappaB. Sci Rep.

[26] Phoomak C, Vaeteewoottacharn K, Silsirivanit A, Saengboonmee C, Seubwai W, Sawanyawisuth K (2017). High glucose levels boost the aggressiveness of highly metastatic cholangiocarcinoma cells via O-GlcNAcylation. Sci Rep.

[27] Kaku H, Goldstein IJ, Oscarson S (1991). Interactions of five D-mannose-specific lectins with a series of synthetic branched trisaccharides. Carbohydr Res.

[28] Pilobello KT, Slawek DE, Mahal LK (2007). A ratiometric lectin microarray approach to analysis of the dynamic mammalian glycome. Proc Natl Acad Sci USA.

[29] Zhao Y, Li J, Wang J, Xing Y, Geng M (2007). Role of cell surface oligosaccharides of mouse mammary tumor cell lines in cancer metastasis. Indian J Biochem Biophys.

[30] Lucena MC, Carvalho-Cruz P, Donadio JL, Oliveira IA, de Queiroz RM, Marinho-Carvalho MM (2016). Epithelial–mesenchymal transition induces aberrant glycosylation through hexosamine biosynthetic pathway activation. J Biol Chem.

[31] Stanley P, Schachter H, Taniguchi N. N-glycans. In: Varki A, Cummings RD, Esko JD, Freeze HH, Stanley P, Bertozzi CR, et al, editors. Essentials of glycobiology. 2nd ed. NY: Cold Spring Harbor; 2009; p. 101–14.

[32] Coomans de Brachene A, Demoulin JB (2016). FOXO transcription factors in cancer development and therapy. Cell Mol Life Sci.

[33] Kannagi R, Yin J, Miyazaki K, Izawa M (2008). Current relevance of incomplete synthesis and neo-synthesis for cancer-associated alteration of carbohydrate determinants--Hakomori’s concepts revisited. Biochim Biophys Acta.

[34] de Leoz ML, Young LJ, An HJ, Kronewitter SR, Kim J, Miyamoto S (2011). High-mannose glycans are elevated during breast cancer progression. Mol Cell Proteom.

[35] Zhang X, Wang Y, Qian Y, Wu X, Zhang Z, Liu X (2014). Discovery of specific metastasis-related N-glycan alterations in epithelial ovarian cancer based on quantitative glycomics. PLoS ONE.

[36] Park HM, Hwang MP, Kim YW, Kim KJ, Jin JM, Kim YH (2015). Mass spectrometry-based N-linked glycomic profiling as a means for tracking pancreatic cancer metastasis. Carbohydr Res.

[37] Tao SC, Li Y, Zhou J, Qian J, Schnaar RL, Zhang Y (2008). Lectin microarrays identify cell-specific and functionally significant cell surface glycan markers. Glycobiology.

[38] Morishima S, Morita I, Tokushima T, Kawashima H, Miyasaka M, Omura K (2003). Expression and role of mannose receptor/terminal high-mannose type oligosaccharide on osteoclast precursors during osteoclast formation. J Endocrinol.

[39] Powers TW, Neely BA, Shao Y, Tang H, Troyer DA, Mehta AS (2014). MALDI imaging mass spectrometry profiling of N-glycans in formalin-fixed paraffin embedded clinical tissue blocks and tissue microarrays. PLoS One.

[40] Zhang P, Wang C, Ma T, You S (2015). O-GlcNAcylation enhances the invasion of thyroid anaplastic cancer cells partially by PI3K/Akt1 pathway. Onco Targets Ther.

[41] Liu Y, Cao Y, Pan X, Shi M, Wu Q, Huang T (2018). O-GlcNAc elevation through activation of the hexosamine biosynthetic pathway enhances cancer cell chemoresistance. Cell Death Dis.

[42] Heath JM, Sun Y, Yuan K, Bradley WE, Litovsky S, Dell’Italia LJ (2014). Activation of AKT by O-linked N-acetylglucosamine induces vascular calcification in diabetes mellitus. Circ Res.

[43] Chen S, Han Q, Wang X, Yang M, Zhang Z, Li P (2013). IBP-mediated suppression of autophagy promotes growth and metastasis of breast cancer cells via activating mTORC2/Akt/FOXO3a signaling pathway. Cell Death Dis.

[44] Yan F, Liao R, Farhan M, Wang T, Chen J, Wang Z (2016). Elucidating the role of the FoxO3a transcription factor in the IGF-1-induced migration and invasion of uveal melanoma cancer cells. Biomed Pharmacother.

[45] Yang JY, Zong CS, Xia W, Yamaguchi H, Ding Q, Xie X (2008). ERK promotes tumorigenesis by inhibiting FOXO3a via MDM2-mediated degradation. Nat Cell Biol.

[46] Liu T, Zhang S, Chen J, Jiang K, Zhang Q, Guo K (2014). The transcriptional profiling of glycogenes associated with hepatocellular carcinoma metastasis. PLoS ONE.

[47] Milde-Langosch K, Karn T, Schmidt M, zu Eulenburg C, Oliveira-Ferrer L, Wirtz RM (2014). Prognostic relevance of glycosylation-associated genes in breast cancer. Breast Cancer Res Treat.

[48] Vojta A, Samarzija I, Bockor L, Zoldos V (2016). Glyco-genes change expression in cancer through aberrant methylation. Biochim Biophys Acta.

[49] Uthaisar K, Vaeteewoottacharn K, Seubwai W, Talabnin C, Sawanyawisuth K, Obchoei S (2016). Establishment and characterization of a novel human cholangiocarcinoma cell line with high metastatic activity. Oncol Rep.

[50] Saentaweesuk W, Araki N, Vaeteewoottacharn K, Silsirivanit A, Seubwai W, Talabnin C et al. Activation of Vimentin is Critical to Promote a Metastatic Potential of Cholangiocarcinoma Cells. Oncol Res. 2017. 10.3727/096504017X15009778205068. 10.3727/096504017X15009778205068 PMC7844738 28762325

[51] Zhu Q, Zhou L, Yang Z, Lai M, Xie H, Wu L (2012). O-GlcNAcylation plays a role in tumor recurrence of hepatocellular carcinoma following liver transplantation. Med Oncol.

[52] Lin C, Wu Z, Lin X, Yu C, Shi T, Zeng Y (2011). Knockdown of FLOT1 impairs cell proliferation and tumorigenicity in breast cancer through upregulation of FOXO3a. Clin Cancer Res.

[53] Park D, Arabyan N, Williams CC, Song T, Mitra A, Weimer BC (2016). Salmonella typhimurium enzymatically landscapes the host intestinal epithelial cell (IEC) surface glycome to increase invasion. Mol Cell Proteom.

[54] Kronewitter SR, An HJ, de Leoz ML, Lebrilla CB, Miyamoto S, Leiserowitz GS (2009). The development of retrosynthetic glycan libraries to profile and classify the human serum N-linked glycome. Proteomics.

