# Supplementary material for: O-GlcNAcylation mediates metastasis of cholangiocarcinoma through FOXO3 and MAN1A1
Source: Oncogene. 2018 Jun 18;37(42):5648–65. doi: 10.1038/s41388-018-0366-1 (PMC6151127; doi:10.1038/s41388-018-0366-1)
Supplement: Supplementary file 1 — Supplementary Figures [file 41388_2018_366_MOESM1_ESM.docx]

**Supplementary Figure** **S1.** The immunohistochemistry using PSA vs. Con A in tumor tissues from CCA patients were compared. PSA could differentiate CCA tissues with non-metastatic vs. metastatic stages. PSA reacted strongly with metastatic stage CCA, weaker with non-metastatic stage CCA; and negative with hepatocytes (H). Con A, in contrast, reacted strongly with both metastatic and non-metastatic CCA, as well as hepatocytes.

**Supplementary Figure** **S2.** Cell aggregation test. To select the *Pisum Sativum* Agglutinin (PSA) concentration, cell aggregation test were performed by various PSA concentrations (0.78 – 100 µg/ml). KKU-213, 4 x 10^4^ cells, were incubated with PSA in 48-well plate for 1 h and the aggregated cells were observed under the microscope.

**Supplementary Figure** **S3.** Inhibited Akt and Erk activation had no effect on O-GlcNAcylation status in CCA cells. The levels of OGP and OGT were determined in **(a)** Akt inhibitor, MK-2206, and **(b)** Erk inhibitor, PD98059, treated cells using western blot analysis.

**Supplementary Figure** **S4.** The PCR products from the real-time PCR of chromatin immunoprecipitation (ChIP) assay were run in the 2% agarose gel electrophoresis. The expected band is 118 base pairs. All of the ChIP samples that immunoprecipitated with anti-FOXO3 showed the single band between 100-200 base pairs. The specific binding of anti-FOXO3 was confirmed by adding IgG isotype control. The band of PCR product could not be detected in the ChIP with IgG isotype control.
